# Customized Appliance Device for Force Detection in Bruxism Individuals: An Observational Study

**DOI:** 10.1155/2022/2524327

**Published:** 2022-06-14

**Authors:** Matteo Pollis, Pietro Maoddi, Marco Letizia, Daniele Manfredini

**Affiliations:** ^1^School of Dentistry, Department of Medical Biotechnology, University of Siena, Viale Mario Bracci, 16, Siena 53100, Italy; ^2^Aesyra SA, c/o EPFL Innovation Park, Building C, Lausanne 1015, Switzerland; ^3^School of Dentistry, Department of Medical Biotechnology, University of Siena, Siena 53100, Italy

## Abstract

**Objective:**

This study aims to test a customised device for detecting contact-related sleep bruxism in adult patients and to show the efficacy of an established biofeedback method incorporated within the device.

**Methods:**

Four volunteers, three of whom suffered from bruxism and one did not, underwent four tests to assess bruxism-related force detection during sleep with concurrent electromyographic recording and to compare SB activity with and without biofeedback stimuli.

**Results:**

The device detected sleep bruxism in bruxer individuals, whilst no activity emerged in the control individual. A correlation between EMG and device signals for bruxism-related events emerged. Moreover, bruxism activity showed a significant decrease on the nights when the biofeedback treatment was applied.

**Conclusion:**

The force-based device can detect appliance-contacting SB events as reliably as EMG recording. Finally, biofeedback stimuli allowed achieving a reduction in the severity and frequency of SB events.

## 1. Introduction

According to the first international consensus definition of 2013, bruxism was defined as a repetitive jaw muscle activity characterised by clenching or grinding of the teeth and/or by bracing or thrusting of the mandible. Bruxism has two circadian manifestations: it can occur during sleep (sleep bruxism, SB) or during wakefulness (awake bruxism, AB) [1]. More recently, in an updated document, an expert panel has redefined bruxism considering the different behaviours observed during sleep and wakefulness. The updated definition suggests that sleep and awake bruxisms are masticatory muscle activities that occur during sleep (characterised as rhythmic or nonrhythmic) and wakefulness (characterised by repetitive or sustained tooth contact and/or by bracing or thrusting of the mandible), respectively. They are not disorders in otherwise healthy individuals [2].

The prevalence rates among adults range from 10% to 13% for SB and 22%–31% for AB [3], whilst it is about 40%–50% in younger populations [3, 4]. However, it must be taken into account that this high variability could depend on lack of standardised assessment methods [5]. As bruxism is no longer considered a disorder but rather a behaviour that can have a harmful, neutral, or protective effect, its assessment plays a fundamental role in determining when it is likely to become a risk (or protective) factor for an underlying disorder [2].

Bruxism assessment considers both noninstrumental and instrumental approaches. The first includes self-report (questionnaires) and clinical examination, which are useful and simple methods for gathering large sample data, but they are not always reliable [2]. Instrumental approaches mainly include electromyography (EMG) and polysomnography (PSG), which cannot easily distinguish between different muscle activities and, above all, do not allow an evaluation of their impact on teeth [2, 6]. Thus, force-based devices have been emerging as useful tools for integrating the study of bruxism, especially considering the possibility of incorporating biofeedback stimuli for management purposes [7]. They consist of modified intraoral splints with force-detecting sensors, which can record occlusal forces and pressure produced during muscle activities. Thanks to the intraoral sensors, force-based devices may integrate EMG-based bruxism detection methods [8]. Nevertheless, there is still poor knowledge on the possibility to distinguish bruxism activities and their different motor patterns (e.g., grinding, clenching, bracing, and thrusting) from other muscle activations [7].

The biofeedback technologies associated with force-based devices (i.e., vibratory stimulus) seem to be a promising approach for interrupting bruxism when needed [7]. Nakamura et al. in a pilot study reported relevant effectiveness of a vibratory stimulus on the suppression of SB [9] by using a force-based splint. However, in line with a recent review [10] and investigation confirming the high effectiveness of vibratory biofeedback [11], more longitudinal studies with a longer observational time and larger patient population are needed to deeply understand this topic.

In this study, a new prototype of a force-based device is proposed. It is custom made on dental models using standard plastic materials for dental splints and contains stretchable capacitive force sensors allocated in the premolar and molar regions, with a geometry designed to differentiate clenching and grinding patterns. Two versions of the device have been tested. A first-generation device is supported by a battery-operated external unit connected to the appliance with a wire, and a second-generation device is completely self-contained, with all the electronics sealed in it. The primary aim of this preliminary study is to test the hypothesis that this force-detection system can do the following:

- Quantify the exerted pressure and produce an output signal allowing to distinguish between different jaw movement patterns
- Detect sleep bruxism in a sample of adult patients and be tolerated by patients using the first-generation prototype
- Detect simulated sleep bruxism episodes comparably to masseter EMG.

In addition, the secondary aim is to show the feasibility and efficacy of an established biofeedback method (vibratory pulse in response to sleep bruxism episodes) incorporated within the appliance.

## 2. Materials and Methods

The study was organized into four different tests, assessing the different aims. A sample of four volunteers (1 male and 3 females; mean age = 27.5 years) three of which were previously diagnosed with sleep bruxism based on the positive answer to the specific oral behaviour checklist question on sleep bruxism and one of which was a nonbruxer. Prior to the study, the control volunteer underwent bruxism evaluation using a force-based device in order to confirm the lack of sleep bruxism activity.

All procedures performed in studies involving human participants were in accordance with the ethical standards and with the 1964 Helsinki Declaration and its later amendments or comparable ethical standards. Informed consent was obtained from all individual participants included in the study.

### 2.1. Test #1: Force Detection during Wakefulness in Laboratory Setting

**Figure 1:**
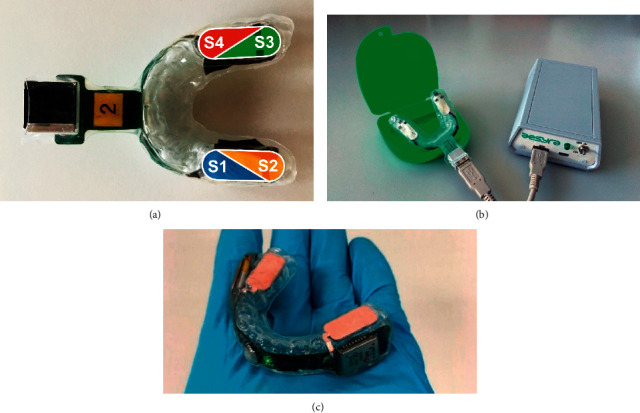
(a) Sensor placement in the prototype bite. (b) First generation prototype used in test #2. (c) Second generation (wireless) prototype used in test #3.

The prototype appliances (first-generation) were custom-made by the Aesyra team (Lausanne, Switzerland) on dental casts of the volunteers, using a standard plastic material for dental splints and containing four stretchable force sensors as well as a minimal version of the flexible electronics (the analogue to digital front-end sensor readout). The sensors were located in the premolar and molar regions of both sides and divided into four areas (S1, S2, S3, and S4) as shown in Figure 1(a). A USB port protruding from the lips provided access to the sensors' data.

Force-displacement measurements with a mechanical test machine were conducted to obtain the response curve (i.e., the force-capacitance relation) of the stretchable sensors and thus allow to quantify the forces exerted on the devices. Water immersion tests were performed as well to verify correct encapsulation and waterproofness.

Volunteers were asked to produce different kinds of jaw movements (clenching, lateral grinding, and forward grinding) that typically occur in bruxism events while wearing the prototype appliance. The digitalised sensor data were read and recorded directly in a notebook. The aim was to assess if the selected sensor design and placement are suitable to quantify the exerted pressure and distinguish between different jaw movement patterns.

### 2.2. Test #2: Force Detection during Sleep in the Natural (i.e., Home) Environment

For this purpose, volunteers were given a mobile device with a test application, which was necessary for the daily downloading of data recorded, and a first-generation prototype appliance to use at home, after being instructed on the use.

The custom-made first-generation prototypes were wired to an external battery-operated unit capable of recording the data and transmitting it to the mobile application (Figure 1(b)).

Each volunteer slept with the appliance, which recorded the bruxism activity in its internal memory. Every morning, the user activated the mobile application, which received and stored the data from the appliance via a wireless connection (Bluetooth low energy). Up to four consecutive nights per each participant were analysed.

Data from the mobile devices were recovered at the end of the tests and analysed by an in-house made software that automatically generated a report for every night of sleep.

The report shows the data gathered from the sensors for the whole night as well as a detailed event-by-event chart. The following parameters were calculated: total number of detected bruxism episodes, bruxism index (number of episodes per hour), average and maximal episode duration, and average episode spacing. For every event, the exerted dental force profile was calculated for the left and right sides of the jaw.

### 2.3. Test #3: Force Detection and Concurrent Electromyography Recording

The second-generation prototype used for this test (Figure 1(c)) derives from the above and is completely self-contained, with all the electronic components encapsulated in the appliance. The battery is recharged wirelessly when the system is not in use (during the day).

The test was performed with a volunteer wearing a second-generation prototype while having, at the same time, electromyography (EMG) electrodes placed on the masseter muscle (Myoware, Advancer Technologies LLC, USA). During the test, the user was asked to produce different kinds of jaw movements (clenching, lateral grinding, and forward grinding) that typically occur in bruxism events, as well as facial movements (head motion and speaking) that may occur involuntarily during sleep. The goal was to assess the capability of the force-detection device to detect bruxism episodes by comparing it to EMG, which is a well-known standard of reference and is used in some commercial bruxism assessment tools.

### 2.4. Test #4: Biofeedback Treatment

The goal of this test was to show the feasibility and the efficacy of an established biofeedback method (vibratory pulse in response to bruxism episodes) with the system. To deliver the vibration, a modified Hexiwear (MikroElektronika DOO, Serbia) smartwatch in Bluetooth communication with the device was used. The average baseline bruxism activity was established for each subject by recording three nights of sleep with the prototype without delivering any pulse with the Hexiwear bracelet. For another five nights of sleep, the Hexiwear bracelet was programmed to emit two mild vibration pulses of 300 ms duration after about 2 seconds from the onset of a bruxism event.

The files with the recordings of all the nights were analysed automatically by the custom software described before to detect the occurrence rate of bruxism events per hour (average bruxism index), the mean bruxism event duration (average episode duration), and the mean duration of bruxism activity per hour (average bruxism activity).

## 3. Results

### 3.1. Test #1

**Figure 2:**
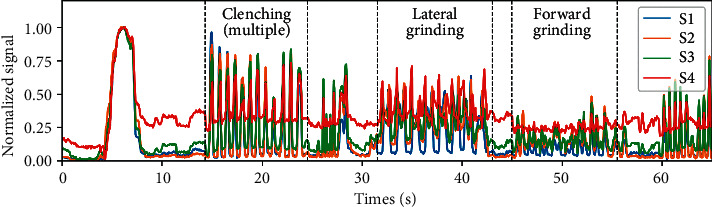
Typical output of the prototype appliances in response to different jaw movements by the subject. Each signal is normalised to the peak of the sustained clenching event occurring at *t* = 6 s.

In the first test, the volunteers were asked to perform different kinds of jaw movements in sequence (i.e., clenching, lateral grinding, and forward grinding). As reported in Figure 2, the tracings referred to the four sensors (S1, S2, S3, and S4) present a visually different pattern for each of the movements, showing that these can be distinguished.

### 3.2. Test #2

In all cases, bruxism activity with contacts on the device could be detected since the first night of use, whilst close to no activity was detected by the device worn by the nonbruxer individual.

**Figure 3:**
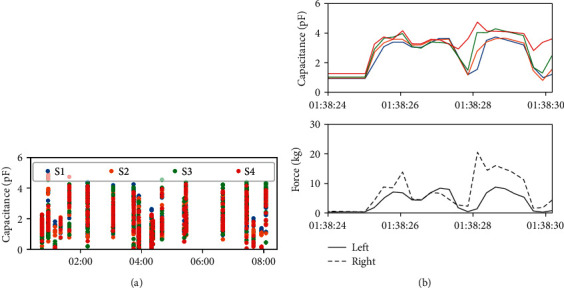
(a) Overview of the whole night of recording, in which the 4-coloured dots represent the signal from each of the 4 sensors in the bite. (b) Example of one of the detected episodes, which shows the dynamics of the bruxism episode and the occlusion forces calculated for the left and right parts of the jaw.

As shown in Figure 3(a), an overview of the whole night was generated, in which the 4-coloured dots represent the signal from each of the 4 sensors in the bite (thus, device contact-bruxism episodes appear as columns in the whole-night plot as each episode lasts 5–20 seconds). A detail of each detected episode is given in Figure 3(b), which shows the dynamics of the bruxism episode and the occlusion forces calculated for the left and right parts of the jaw.

**Figure 4:**
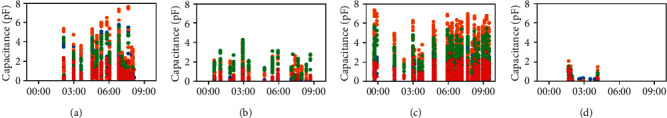
Comparison of a full night of recording for the four test subjects. The 4-coloured dots represent the signal from each of the 4 sensors in the bite. Activity in subjects affected by bruxism (a–c) and a not-affected one (d) is shown.

In addition, the comparison of a full night of recording for the four subjects was performed. In Figure 4, the 4-coloured dots represent the signal from each of the four sensors in the appliance. The difference in activity between subjects affected by bruxism (a–c) and a not-affected one (d) is clearly visible. Differences in the number, rate, duration, and intensity of bruxism episodes can be appreciated among the subjects affected by bruxism as well.

### 3.3. Test #3

**Figure 5:**
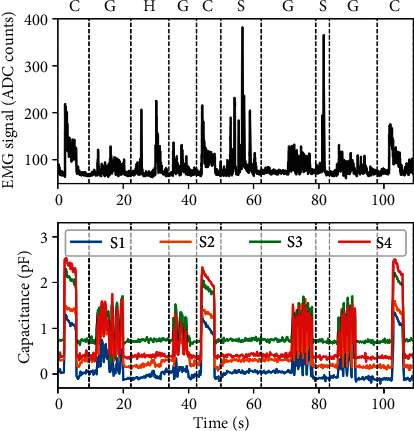
Correlation between EMG and force-sensing appliance signals for bruxism-related events (clenching (C) and grinding (G)). Rejection of false positives is shown (EMG signals due to speaking (S) and moving the head (H)).

A visual comparison between the masseter EMG signal versus the force sensor traces (Figure 5) shows a good correlation between the two techniques for bruxism-related movements (i.e., clenching (C) and grinding (G)). At the same time, bruxism-unrelated motion (i.e., head movement (H) and speaking (S)) are not visible in the force sensors signal, hinting at good specificity for tooth-contact events.

### 3.4. Test #4

**Figure 6:**
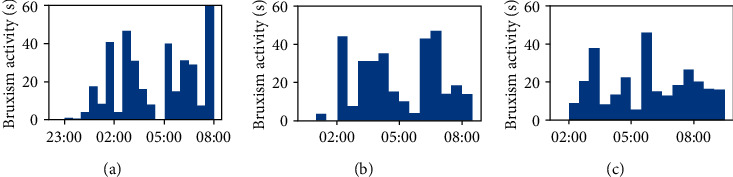
(a–c) Establishment of the baseline bruxism severity from 3 nights of recording without biofeedback.

The average bruxism index, the average episode duration, and the average bruxism activity were calculated by analysing three nights of recording of sleep without any biofeedback. The calculated values (mean ± standard deviation) were 6.8 ± 0.8 episodes/h, 7.0 ± 3.3 s, and 45.6 ± 15.7 s/h, respectively (Figure 6).

**Figure 7:**
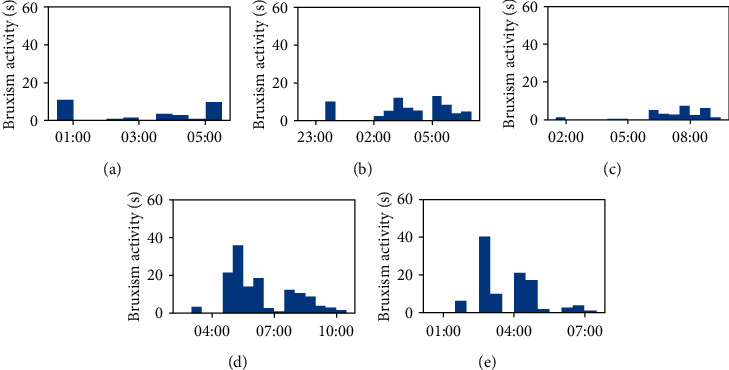
(a–e) Establishment of the bruxism severity during 5 nights of recording with the biofeedback treatment.

On the other hand, the same three parameters were assessed after five nights by delivering biofeedback treatment. The mean bruxism index was 3.0 ± 0.8 episodes/h, the average episode duration was 3.2 ± 1.3 s, and the average bruxism activity was 10.5 ± 6.5 s/h (Figure 7).

A remarkable decrease in the severity of bruxism was observed on the nights where the biofeedback treatment was applied, with bruxism index, episode duration, and bruxism activity being reduced, respectively, by 56%, 53%, and 77% on average. However, an unpaired *t*-test shows that while the reduction in bruxism index and bruxism activity is significant (*p* < 0.05 in both cases), the reduction in average episode duration is not quite significant (*p*=0.054).

## 4. Discussion

In recent years, the bruxism definition has been going through several changes. The construct of bruxism as a behaviour that may have either a detrimental or a protective effect but is not necessarily a disorder has opened up a new era for clinical research, with a focus on the need to establish parameters of biological variability. Bruxism assessment is based on different instrumental (e.g., PSG and EMG) and noninstrumental approaches (e.g., questionnaires, clinical evaluation, and ecological momentary assessment), each of which has its peculiar advantages [2]. Currently, since the bruxism definition embraces muscle activities possible, but not necessarily, involving teeth contact, research on the distinction of different bruxism types might be enhanced by the use of force-measurement-based devices. This approach may also be useful to implement biofeedback technology for bruxism management purposes [12]. On the other hand, there is still a lack of knowledge on this topic, especially concerning the possibility to detect the different bruxism motor patterns and the effects of biofeedback.

In this study, a new force-based device was tested. It contains stretchable capacitive force sensors allocated in the premolar and molar regions. A first-generation device (connected to a battery-operated external unit) was first used to test the software, and then a self-contained second-generation device (wireless) was manufactured and tested for comparison with EMG findings and for the effectiveness of biofeedback (i.e., vibratory stimulus). All in all, the main scopes were to evaluate the detection of bruxism episodes associated with contacts on the device (first aim) and the feasibility and efficacy of the biofeedback method (second aim).

Findings showed that the tested device was able to detect different motor patterns related to bruxism during wakefulness, such as clenching, lateral grinding, and forward grinding (first test). The so-detected bruxism events in the four volunteers were recorded during sleep to assess the differences in the number, rate, duration, and intensity of bruxism episodes (second test). The differences between the three individuals who were previously assessed with bruxism and the control subject were notable. The second-generation device was as reliable as the EMG in recognizing bruxism events and discarding muscle activities not related to bruxism (third test). The vibratory biofeedback system determined a decrease in the severity of bruxism with bruxism index, episode duration, and bruxism activity, respectively, by 56%, 53%, and 77% on average (fourth test).

Oral appliances are a bruxism management option to prevent or reduce tooth wear due to teeth contact and grinding habits, even if their influence in terms of bruxism detection and reduction has never been consistently supported [13]. Concerning the bruxism activities with teeth contact, determining the types of motor patterns which occur in bruxism patients is based on the signs of wear on the teeth or appliance surface, and its detection requires a good professional experience [7]. At present, no standardised protocols to assess it has been proposed. As stated by Baba et al., the use of an intra-appliance force detector has good reliability for bruxism detection [14]. In the majority of force-based devices, sensors are packed in the canines or premolars regions, not allowing balanced forces detection along the whole dental arch. In accordance with Gao et al., full coverage of dentition in combination with multisite stress sensors can guarantee a better detection [7]. In the current study, force sensors have been located in both molars and premolars regions and can quantify the exerted pressure and distinguish between different jaw movement patterns, as suggested by the preliminary results shown in this investigation.

The diagnostic accuracy of force-based devices in the SB assessment has been raising considerable interest in literature. As asserted in the international consensus, EMG recordings during sleep provide key evidence of sleep bruxism [1, 2, 15] and may also be integrated with other measures to study the sleep correlating via cardiorespiratory monitoring or full polysomnography. However, the number of events (e.g., burst) and their duration are usually just summed up and expressed per hour of sleep, thus offering only a partial representation of the amount and pattern of muscle activity. Other EMG outcome measures, such as power (area), peak amplitude, interval duration between activities, and the distinction between grinding and clenching, could be included [16–18]. Casett et al. [19] and Manfredini et al. [20] suggested that portable devices have acceptable values of specificity and sensitivity with respect to PSG criteria, but few data are available on force detectors. Takeuchi et al. compared the occlusal pressure signal and EMG activity and asserted that the force-based devices reported excellent data on grinding movements [21]. However, EMG had a better signal for clenching [12]. The authors' findings confirmed this evidence. EMG detected all motor patterns related to bruxism and discriminated from other activities (e.g., speaking and moving the head) with higher accuracy for clenching than for grinding. On the contrary, the prototype did not detect activities not related to bruxism, as reported in Figure 5. A possible explanation for this outcome is the absence of dental contact during speaking and moving the head activities.

Consistent with Beddis et al. in their overview on sleep bruxism, biofeedback treatment aims to provide immediate information to the patient about their behaviour, enabling its reduction [22] through many techniques (e.g., EMG with auditory and vibratory stimulatory feedback). Gu et al. proved that biofeedback therapy may be effective for mild bruxers when compared with occlusal splint therapy [8]. In addition, Bergmann et al., testing a full-occlusion biofeedback splint on SB patients in a randomized control clinical trial, reported higher effectiveness in reducing SB at the subconscious level and in achieving improvements in global pain perception than in the control group [11]. The present study supported these outcomes. On the other hand, a review by Wang et al. concluded that there was the absence of evidence to support the use of biofeedback technology in SB treatment [23]. Moreover, they suggested that trials on larger samples and adopting homogeneous outcome indexes are recommended.

Jokubauskas et al., in a recent systematic review, confirmed these reports, asserting that despite the positive results of many studies, evidence remains uncertain due to a limited amount of works included in the meta-analysis [10]. Besides, although some biofeedback modalities, such as contingent electrical stimulation (CES), showed a significant effect on the reduction in SB-related EMG events, evidence of long-term effects is lacking.

All in all, this preliminary study adds some information to the paucity of work on force-based appliance devices. The ability to distinguish different types of movements offers many potential benefits, such as facilitating the estimation of forces exerted during contact-related SB by calibrated sensors, reducing the likelihood of artefacts in the detection of motor patterns, and providing valuable information on the true nature of contacts involved and the variations seen within and between individuals [12]. On the other hand, several limitations are present. Firstly, the small sample size with mixed-gender subjects is a significant limitation of the study, not providing a sufficient basis for any statistical inferences. The results are just indicative of potential correlation with EMG recording. In addition, since two distinct aims are assessed, two different study designs should have been performed. As suggestions for future studies, a comparison with PSG, considered one of the main SB evaluation instruments so far, is highly recommended. Many authors focus on the concept of scoring the whole spectrum of the masticatory muscle activity (MMA) rather than the scoring of SB events, in order to better define the different manifestations of SB activity [24, 25]. For instance, in patients affected by obstructive sleep apnoea (OSA), SB could have a protective role by ending the apnoea event [2]. Therefore, the use of the appliance and their consequences on sleep patterns should be deeply investigated.

## 5. Conclusion

The present study showed that a new force-based appliance may quantify the exerted pressure by the masticatory muscles and distinguish between different jaw movement patterns which occur during bruxism. It can detect appliance-contacting SB events with a good correlation with EMG recordings and exclude motor patterns not related to SB. Finally, biofeedback stimuli provided by an associated smartwatch allowed achieving a reduction in the severity and frequency of SB events.

## Data Availability

The data underlying this article will be shared upon reasonable request to the corresponding author.
